# Viscosity Characterization of PDMS and Its Influence on the Performance of a Torsional Vibration Viscous Damper Under Forced Hydrodynamic Loading

**DOI:** 10.3390/ma19030490

**Published:** 2026-01-26

**Authors:** Andrzej Chmielowiec, Adam Michajłyszyn, Justyna Gumieniak, Sławomir Woś, Wojciech Homik, Katarzyna Antosz

**Affiliations:** 1 The Faculty of Mechanics and Technology, Rzeszow University of Technology, Kwiatkowskiego 4, 37-450 Stalowa Wola, Poland; achmie@prz.edu.pl (A.C.); a.michajlysz@prz.edu.pl (A.M.); j.gumieniak@prz.edu.pl (J.G.); 2 The Faculty of Mechanical Engineering and Aeronautics, Rzeszow University of Technology, Powstańców Warszawy 12, 35-029 Rzeszów, Poland; wosslawomir@prz.edu.pl (S.W.); whomik@prz.edu.pl (W.H.)

**Keywords:** polydimethylsiloxane (PDMS), dynamic viscosity, hydrodynamic loading, torsional vibration viscous damper, hydrodynamic lubrication theory

## Abstract

This study presents the experimental and model-based characterization of polydimethylsiloxane (PDMS) as a damping medium in a torsional vibration viscous damper. Particular emphasis is placed on the influence of the PDMS viscosity on the dynamic response of the damper under variable hydrodynamic loading generated by torsional vibrations of the system and the mass of the inertia ring. Investigations were conducted over a wide range of kinematic viscosities, enabling the identification of damper operating regimes and the assessment of lubricating film stability. The developed mathematical model, based on hydrodynamic lubrication theory, describes the relationships between the PDMS viscosity, the relative angular velocity, and the eccentricity of the inertia ring. Experimental results confirm the model’s ability to predict transitions between stable, unstable, and boundary operating modes of the damper. The proposed approach enables the functional, system-level characterization of PDMS under hydrodynamic loading conditions within a torsional vibration damper. In this framework, the rheological properties of PDMS are directly linked to the dynamic response and operational stability of the mechanical system.

## 1. Introduction

The characterization of engineering materials under real operating load conditions constitutes one of the key challenges of modern materials engineering. In the case of polymeric materials such as polydimethylsiloxane (PDMS), the dependence of rheological properties on load, temperature, and flow conditions plays a crucial role. In dynamic applications, where the material simultaneously serves as both a lubricating and damping medium, classical viscosity characterization must be complemented by an analysis of the material’s response to dynamic and hydrodynamic excitations.

Within this context, torsional vibrations are considered some of the most critical dynamic phenomena occurring in mechanical systems, particularly in the drive systems of machines and vehicles powered by internal combustion engines. They are generated as a result of variable torque loads, induced both by the non-uniform operation of the engine and the changing loads transmitted through working components. Their presence is associated with a range of adverse effects—from increased noise and vibration levels in the operator’s cabin, through the elevated wear of mechanical elements, to the risk of severe structural damage. The issue of noise, in the context of insufficient torsional vibration damping and engine operation within resonant speed ranges, has been discussed by Barton and Fieldhouse [1], as well as Qatu [2]. The impact of improperly selected dampers on the generation of harmful acoustic vibrations has also been highlighted by Lopez et al. [3].

One of the most effective solutions for mitigating the impact of torsional vibrations is considered to be the torsional vibration viscous damper. These devices are operated based on the properties of a viscous fluid, in which an inertia ring is immersed and allowed to move relative to the housing. This relative motion is associated with the shearing of the oil layer, resulting in the dissipation of vibrational energy in the form of heat. As a consequence, the amplitude of vibrations is effectively reduced, and critical components of the mechanical system are protected from structural damage [4]. Classical constructions of torsional vibration viscous dampers have been thoroughly described by Balykova and Rode [5], as well as Schmitz and Smith [6]. Practical industrial applications of such dampers have been presented by Braund [7] and Windhofer et al. [8]. An analysis of internal phenomena occurring within the damping fluid has been conducted by Venczel and Veress [9].

The first applications of torsional vibration viscous dampers were recorded in the early 20th century and were associated with the reduction of camshaft vibrations in submarine engines with a power output of 300 horsepower [4,10]. Currently, such dampers are widely used in industrial engines, military vehicles, agricultural machinery, rail vehicles, and marine propulsion systems [11,12]. Their importance has been continuously emphasized in the context of increasing demands for durability, operational reliability, and user comfort [13,14,15].

Progress in research on torsional vibration viscous dampers has been focused on enhancing their damping efficiency and improving their adaptability to variable operating conditions. The influence of high-viscosity silicone oil properties on the dynamic characteristics of dampers has been analyzed by Kim et al. [16], and limitations of conventional computational models have been identified. A hybrid construction, combining a rubber and fluid damper to improve the effectiveness of torsional vibration control in crankshaft systems, has been proposed by Sezgen and Tinkir [17]. A thermo-mechanical analysis of damper performance, based on a numerical model developed in Comsol and validated through experimental testing on a six-cylinder diesel engine, has been conducted by Chen et al. [18].

The effectiveness of torsional vibration viscous dampers is determined not only by their design but also to a significant extent by the lubrication conditions, which define the type of friction occurring within the device—either wet or dry. Wet friction, characterized by the presence of a thin yet continuous lubricating film between the housing and the inertia ring surface, is considered critical for the durability and operational reliability of the damper. It has been demonstrated by Bhushan [19] that the presence of a lubricant film reduces friction, prevents surface wear, and lowers the operating temperature. In contrast, it has been shown by Zhang et al. [20] that inadequate lubrication may lead to microcrack formation, overheating, and premature failure of the system.

Lubrication stability has been widely recognized as a critical factor governing friction, wear, and durability in various mechanical systems, including bearings and geared transmissions [21,22,23,24,25,26]. Previous studies have shown that lubricant degradation, insufficient film thickness, and improper surface engineering may lead to transitions toward boundary or dry friction regimes, resulting in accelerated wear and failure [27,28].

Advanced surface engineering solutions, such as micro-texturing and low-friction coatings, have been shown to improve lubrication stability under boundary conditions; however, their application is system-specific and does not eliminate the fundamental dependence of damper operation on the oil viscosity and film thickness [29,30,31,32,33,34,35].

Torsional vibration viscous dampers, like other mechanical devices, are subject to wear and failure, often caused by damping fluid degradation, oil leakage, or insufficient maintenance. It has been indicated by Yang et al. [36] that oil loss leads to uncontrolled vibrations and elevated temperatures, which consequently reduce the damping efficiency. The effects of insufficient damping fluid have been investigated by Chmielowiec et al. [37], who showed that an inadequate oil volume results in the transition to a dry friction regime and increased component wear.

Additionally, the presence of contaminants such as dust, water, or metal particles can significantly deteriorate the lubricating properties of the oil [38]. These contaminants are most often introduced through housing leakage or extended operation without oil replacement. The importance of temperature in relation to the viscosity change of the damping fluid has been emphasized by Chmielowiec et al. [39]. As the oil degrades, a gelation phenomenon is observed, leading to the formation of a solid mass and the blockage of inertia ring movement [40,41].

Damper reliability may further be compromised by oil contamination, thermal degradation, and corrosion effects, which accelerate wear and reduce the damping efficiency if not properly monitored and mitigated [12,42,43].

To prevent failures, the operating conditions of torsional vibration viscous dampers must be regularly monitored, proper maintenance must be performed, and appropriate materials must be selected [44]. Only a comprehensive approach—where the damper is treated as an integrated component of the drivetrain system—allows long-term and reliable operation to be achieved.

The aim of this study is the experimental and model-based characterization of PDMS as a working material in a torsional viscous vibration damper, with particular emphasis on the influence of viscosity on the hydrodynamic lubrication conditions and the operational stability of the damper. The material response to loads generated by torsional vibrations of the system and by the weight of the inertia ring was analyzed. This approach enables a direct correlation between the material properties of PDMS and the dynamic behavior of the mechanical system, constituting a significant contribution to the development of advanced characterization methods for functional materials.

To achieve this objective, the damper operation model proposed in this study is based on hydrodynamic lubrication theory and on an analogy to bearing performance. This modeling framework enables the analysis of physical phenomena occurring within the damper—in particular, the interactions between the damping fluid and the frictional surfaces under variable load and temperature conditions.

## 2. Materials and Methods

The applied research methodology was designed to enable the comprehensive characterization of the viscosity-related properties of PDMS under real hydrodynamic loading conditions occurring in a torsional vibration viscous damper. The combination of experimental investigations and mathematical modeling allows for the identification of relationships between material viscosity, relative motion parameters, and the stability of the lubricating film. This approach makes it possible to treat the damper as an experimental system for the evaluation of the behavior of a polymeric material under dynamic loading.

**Figure 1:**
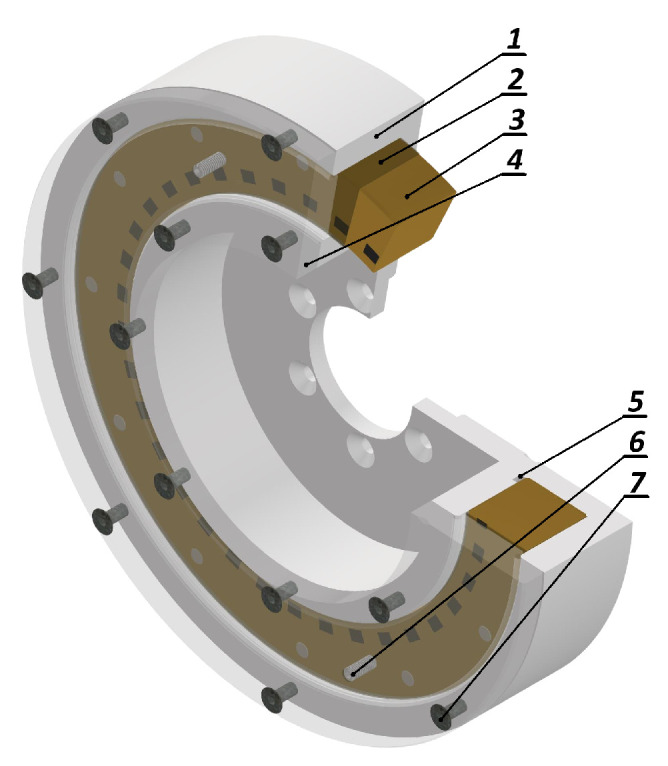
Viscous torsional vibration damper: (1) damper housing, (2) silicone oil, (3) inertia ring, (4) cover, (5) oil channel, (6) filling hole, (7) screw for assembly of the damper.

A torsional vibration viscous damper is a device that utilizes viscous friction within a fluid medium to dissipate the energy of vibrations generated by variable torque excitation. The damper consists of a cylindrical casing containing an inertia ring that is free to move within the limits imposed by the structural geometry (Figure 1). The annular space between the ring and the casing is filled with silicone oil of appropriate viscosity. When torsional vibrations are transmitted to the damper housing, relative motion arises between the casing and the inertia ring, generating viscous shear stresses within the fluid. As a result, vibrational energy is dissipated in the form of heat and transferred to the surroundings. Reliable and long-term operation of the damper is closely associated with the absence of direct contact between the casing and ring surfaces, which ensures stable hydrodynamic lubrication conditions over an extended service life.

### 2.1. Materials

A typical torsional vibration viscous damper is made from high-strength structural steel. However, for the purposes of analyzing its behavior, it was necessary to construct it from non-ferromagnetic materials. For this reason, the decision was made to create its casing in two parts: the housing was derived from aluminum alloy PA6/2017 (Table 1) and the cover from transparent acrylic PMMA. The inertia ring was constructed from bronze B101 (Table 2). The change in materials used for the damper housing and the inertia ring was crucial for the feasibility of the studies. Non-ferromagnetic materials allowed for the installation of magnets and motion registration using Hall sensors, while the transparent PMMA cover enabled the use of infrared sensors. It should also be noted that the use of bronze B101 for the inertia ring allowed for the preservation of its most essential feature: its inertia. The use of a material with a density 14% higher than steel guarantees that the moment of inertia of this component will also be 14% greater compared to its steel counterpart. This was a value that was acceptable from the perspective of the planned experiments.

**Figure 2:**
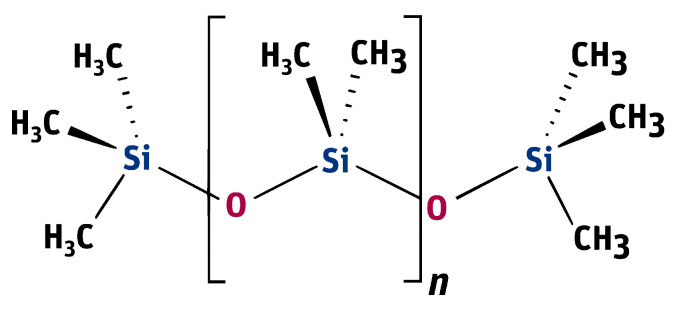
The structural formula of the silicone oil molecule—polydimethylsiloxane [39].

In the present study, polydimethylsiloxane (PDMS, Figure 2) is treated as a functional material whose rheological and lubricating properties determine the dynamic response of a torsional vibration viscous damper. In contrast to the classical approach, in which the viscosity of the damping medium is considered merely an operational parameter, a material-oriented perspective is adopted, focusing on the characterization of PDMS’ behavior under variable hydrodynamic loading resulting from torsional vibrations and the action of the inertia ring mass.

The damper was filled with PDMS silicone oil (Clearco Products Co, Inc., Willow Grove, PA, USA, Table 3). For the experiments, silicone oils with a very wide viscosity range were selected. This approach made it possible to conduct 6 experiments, which demonstrated how critical the viscosity is for the proper functioning of a viscous torsional vibration damper. Silicone oils with viscosities reaching up to 1 m^2^s^−1^ are used to fill dampers. The degree of oil contamination and its viscosity form the basis for determining whether a damper can be approved for operation or needs regeneration [45]. The most commonly used damping material is polydimethylsiloxane containing methyl groups, fulfilling the general requirements imposed on materials intended for vibration damping [46,47].

The properties of PDMS, resulting from its structure, enable the use of this polymer in automotive and industrial applications. As a damping substance, PDMS dissipates vibrations primarily through intermolecular friction. The molecules of this polymer compress and stretch against each other, allowing the formation of strong elastic polymer networks that effectively absorb and dissipate mechanical energy. The vibration amplitude is reduced, and the system becomes more stable due to the conversion of kinetic vibration energy into heat.

PDMS also provides wet friction by forming a lubricating layer between the damper surfaces. This contributes to the stability of the damping process, reduces mechanical wear or overheating, and ensures the long-term, failure-free operation of the device. Wet friction is therefore crucial during damper operation. The reliability of dampers under harsh working conditions—such as humidity, high temperatures, and varying loads—is closely related to the properties of PDMS, such as thermal and chemical stability and resistance to degradation. The properties of polysiloxanes are described, among others, by Balykova and Rode [5], Mark et al. [48], and Liu et al. [49].

One of the main indicators of the quality and performance of a damping medium is its viscosity. Its value depends on the shear rate and the operating temperature of the oil and is related to its aging process. Proper analysis of the properties of silicone oil allows for the correct design of viscous dampers and their subsequent servicing. According to Lewicki et al. [50], Garcia-Garrido et al. [51], and Andra and Spurk [52], understanding the relationship between the structure and properties of polysiloxanes makes it possible to predict their behavior under specific conditions and, consequently, to determine the performance and durability of dampers.

### 2.2. Research Setup

**Figure 3:**
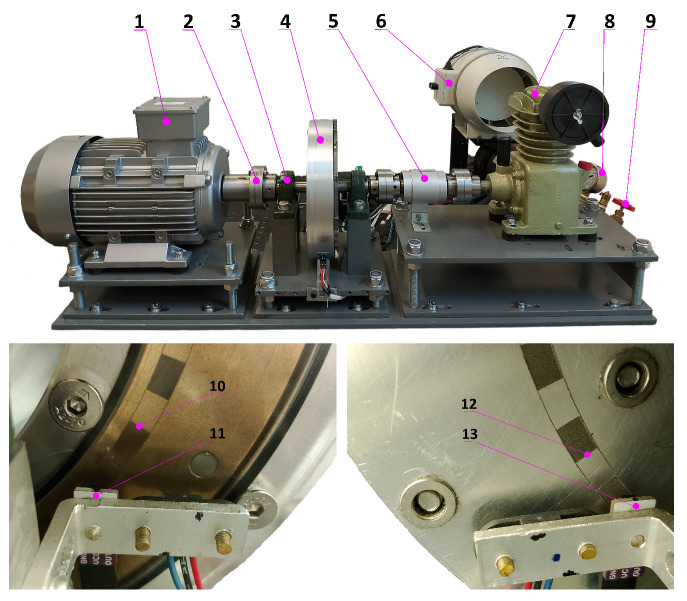
Measurement setup: (1) electric motor with adjustable rotational speed, (2) flange coupling, (3) bearing, (4) torsional vibration viscous damper, (5) torque meter, (6) fan, (7) single-piston compressor, (8) manometer, (9) load adjustment valve, (10/12) light and dark fields on the inertia ring/housing, (11/13) infrared sensor on the inertia ring/housing side.

The measurement setup is presented in Figure 3. It is equipped with a squirrel-cage motor with power of 3.5 kW and a maximum rotational speed of 2800 rpm, which is regulated using a dedicated controller. To generate torsional vibrations, a single-piston compressor cooled by a fan with an adjustable flow is used. All components of the system are connected using flange couplings, which ensure the unchanged transmission of torsional oscillations, a crucial aspect for the nature of the conducted experiments. The load applied to the compressor can be adjusted using a valve, with its stability controlled by a manometer. The torsional moment occurring on the shaft is recorded using a digital torque meter with a maximum sampling frequency of 2600 Hz, which allows for the determination of the amplitude–frequency characteristics of the tested compressor.

**Figure 4:**
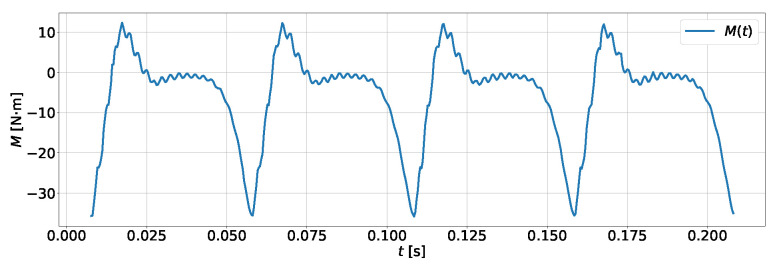
The torque generated by the compressor operating at a frequency of 20 Hz (1200 rpm) and under a load of $10^{6}$ Pa.

The purpose of the developed setup is to induce torsional vibrations on the shaft connecting the electric motor and the compressor. These vibrations are directly transferred to the damper, which is responsible for dissipating them. It should be emphasized that the presence of torsional vibrations is a necessary condition for the active operation of the damper, as it is these vibrations that cause the relative motion of the inertia ring with respect to the casing. Figure 4 presents a graph of the torque M(t) generated by the compressor during operation at a frequency of 20 Hz (1200 rpm) and a pressure in the accumulator tank of $10^{6}$ Pa. The variability in the torque M(t) in the designed setup is periodic, which allows for spectral analysis.

**Figure 5:**
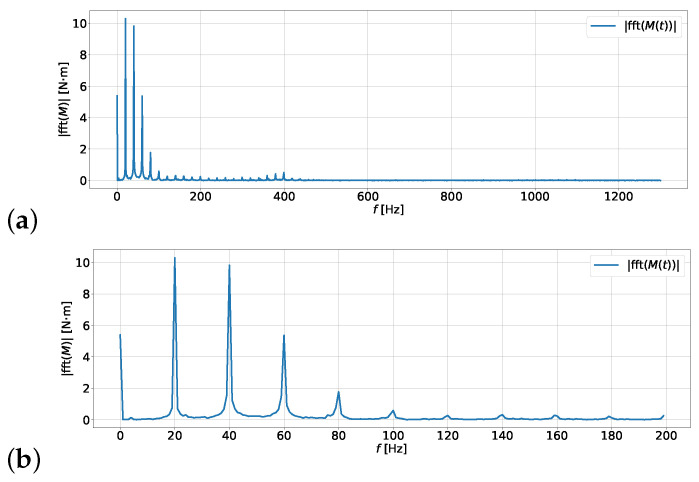
One-sided amplitude spectrum of the torque generated by the compressor operating at a frequency of 20 Hz (1200 rpm) and under a load of $10^{6}$ Pa: (**a**) plot of the complete one-sided spectrum, (**b**) plot of the one-sided spectrum for frequencies from 0 to 200 Hz.

Figure 5 presents the amplitude spectrum plots for the signal M(t). Plot (a) shows the complete one-sided spectrum, while plot (b) narrows the domain to frequencies not exceeding 200 Hz. The presented graph clearly shows that the main frequencies causing vibrations are 20, 40, 60, and 80 Hz. There is also a significant constant component, but it does not affect the damper’s operation, as it does not generate torsional vibrations.

### 2.3. Measurements

The relative motion of the inertia ring and the casing was measured using a sensor consisting of an emitter and a receiver of infrared radiation. Markers were burned onto the casing and the inertia ring at the same diameter, with 36 alternating light and dark fields. The measurement of the geometric dimensions of the damper and its components was carried out using a Mitutoyo Crysta ApekV 544 measuring machine.

The thermal state of the system was monitored using a pyrometer by measuring the surface temperature of the damper housing. During all measurement series, the housing temperature remained within the range of 22–26 °C. This narrow temperature variation ensured stable operating conditions and minimized the influence of thermal expansion and viscosity changes on the obtained results. Consequently, temperature-related effects were not considered a dominant factor influencing the observed variations in angular velocity and oil film thickness.

**Figure 6:**
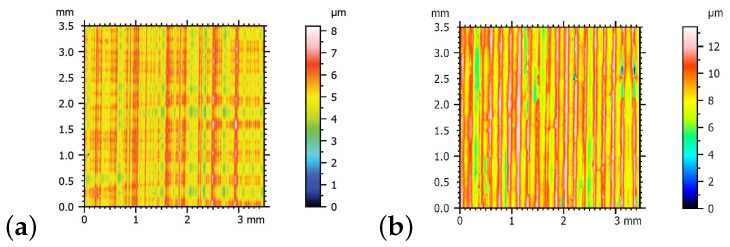
Topographic maps of the contact surfaces of the viscous damper before the tests for (**a**) damper housing, (**b**) inertia ring.

The surface roughness measurements were carried out using a contact profilometer, the i-series PRP by Taylor Hobson. The profilometer was equipped with a measurement head featuring an inductive sensor, with a maximum roughness height measurement range of up to 2 mm and a vertical resolution of up to 1 nm. The measurements were performed on an area of 3.5 mm by 3.5 mm, with a step size of 5 μm. Example topographic maps of the surface before the tests are presented in Figure 6. These are typical surfaces obtained after the turning operation. The height parameters of the surface roughness were described using the parameters Ra, Rz, and Rzx according to ISO 25178 [53]. The roughness parameters for the casing surface were Ra 0.5 μm, Rz 3.24 μm, and Rzx 3.78 μm, while, for the inertia ring surface, they were Ra 1.28 μm, Rz 6.08 μm, and Rzx 7.31 μm.

Due to the high cost and complexity of the experimental setup and the time constraints of the test campaign, a single measurement series was performed for each of the six investigated oil viscosity values. Consequently, a classical repeatability assessment based on multiple independent repetitions of the same test (inter-test repeatability) was not feasible. However, each experiment involved the continuous time-domain acquisition of the measured signals, which enabled the evaluation of measurement uncertainty and the analysis of the temporal variability of the instantaneous angular velocity ω(t) within a single experiment.

## 3. Theory and Calculations

### 3.1. Mathematical Model of the Inertia Ring Equilibrium

**Figure 7:**
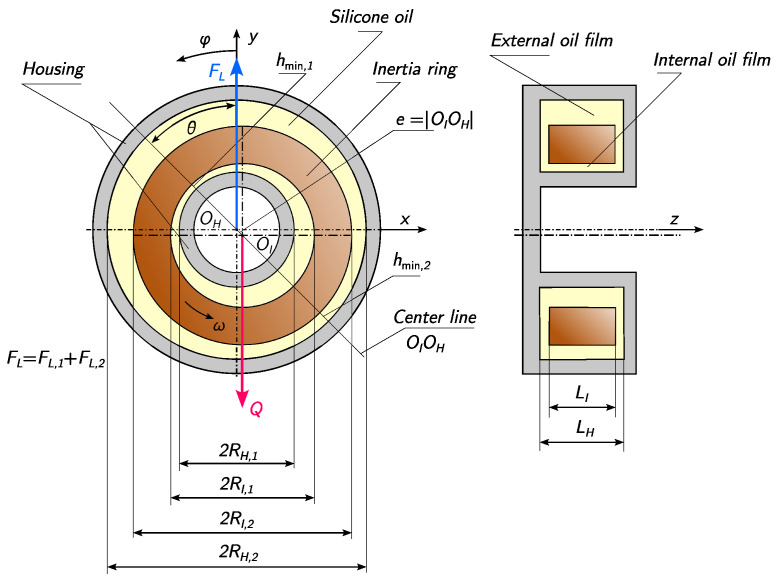
Diagram of the viscous torsional vibration damper showing the dynamic equilibrium state, in which the inertia ring moves relative to the housing with a specified angular velocity ω [54].

For the purposes of the present study, the equilibrium model of the inertia ring, presented in [37], was used. This model assumes that, at a constant relative speed between the housing and the inertia ring, $\omega =|\omega_{H}-\omega_{I}|$, the position of the ring suspended in silicone oil with a dynamic viscosity η remains stable, as shown in Figure 7. It is assumed that

- $R_{I,1},R_{I,2}$ are the inner and outer radii of the inertia ring, respectively;
- $R_{H,1},R_{H,2}$ are the inner and outer radii of the housing, respectively;
- $L_{I},L_{H}$ are the heights of the inertia ring and the housing, respectively;
- $c_{1}=R_{I,1}-R_{H,1},c_{2}=R_{H,2}-R_{I,2}$ are the inner and outer radial clearances, where $c_{1}<c_{2}$;
- *Q* is the weight of the inertia ring;
- $F_{L}$ is the hydrodynamic buoyancy force balancing the weight of the inertia ring, which consists of the hydrodynamic forces from the inner oil film ($F_{L_{1}}$) and the outer oil film ($F_{L_{2}}$);
- θ is the angle by which the line connecting the centers $O_{I}O_{H}$ of the inertia ring and the housing deviates from the horizontal;
- $e=|O_{I}O_{H}|$ is the eccentricity of the inertia ring and the housing.

It should be noted that the condition $c_{1}<c_{2}$ is a design constraint. This ensures that the contact surface between the inertia ring and the housing will be the internal surface. Additionally, the following quantities are defined:

- $R_{i}=\frac{1}{2}\left(R_{I,i}+R_{H,i}\right)$ for i∈1,2 as the average inner radius (i=1) and outer radius (i=2);
- $\varepsilon_{i}=e/c_{i}$ for i∈1,2 as the relative eccentricity for the inner radial clearance (i=1) and outer radial clearance (i=2);
- $h_{\min,i}=c_{i}-e$ for i∈1,2 as the minimum oil film thicknesses in the inner layer (i=1) and outer layer (i=2), respectively.

At this point, it should be emphasized that the values of $\varepsilon_{1}$ and $\varepsilon_{2}$ are not independent. Since the eccentricity *e* is a common quantity for both, there is a relationship between them:

(1) $$\begin{array}{c} \varepsilon_{1}c_{1}=\varepsilon_{2}c_{2}. \end{array}$$

Under these assumptions, the equilibrium condition of the inertia ring, according to [37], is expressed by Equations (2) and (3).

(2) $$\begin{array}{cc} Q\cos\theta & =12\cdot \eta \cdot \omega \cdot L_{I}\sum_{i=1}^{2}\frac{\varepsilon_{i}^{2}R_{i}^{3}}{\left(2+\varepsilon_{i}^{2}\right)\left(1-\varepsilon_{i}^{2}\right)c_{i}^{2}}, \end{array}$$

(3) $$\begin{array}{cc} Q\sin\theta & =6\pi \cdot \eta \cdot \omega \cdot L_{I}\sum_{i=1}^{2}\frac{\varepsilon_{i}R_{i}^{3}}{\left(2+\varepsilon_{i}^{2}\right){\left(1-\varepsilon_{i}^{2}\right)}^{1/2}\;c_{i}^{2}}. \end{array}$$

The introduced relationships allow for a direct connection between the relative angular velocity ω and the relative eccentricity $\varepsilon_{1}$. The latter quantity is particularly important from the standpoint of the contact of the inner surfaces of the inertia ring and the housing. Since $c_{1}<c_{2}$, in fact, only the inner surface can experience wear due to friction. The quantity that allows for determining whether the damper operates under hydrodynamic or mixed friction conditions is $h_{\min,1}=c_{1}\left(1-\varepsilon_{1}\right)$. To this end, it is necessary to experimentally determine the angular velocity ω, based on which $\varepsilon_{1}$ can then be analytically determined and, consequently, $h_{\min,1}$. Therefore, a research setup was designed to investigate the relative angular velocity between the inertia ring and the housing.

The damping oil is modeled as an incompressible Newtonian fluid with temperature-dependent viscosity η=η(T). Non-Newtonian effects and pressure-dependent rheological behavior are neglected. The hydrodynamic pressure in the oil film is assumed to be non-negative, with a uniform pressure distribution in the radial direction and constant ambient pressure. The oil flow is treated as laminar, with a no-slip condition at the solid boundaries and a dominant axial flow component [54].

### 3.2. Numerical Determination of the Relative Eccentricity of the Internal Oil Film

Experimental measurements of the average relative angular velocity are a key element in the process of modeling the oil film thickness. The Formulas (1)–(3) allow for defining the equation $F(\omega ,\varepsilon_{1})=0$, whose solutions determine the relationship between the relative angular velocity ω of the housing and the inertia ring and the value of the relative eccentricity $\varepsilon_{1}$. A series of simple algebraic transformations allows the function *F* to be determined and written in the following form:

(4) $$\begin{array}{cc} F(\omega ,\varepsilon_{1}) & =Q^{2}-{\left(6\cdot \eta \cdot \omega \cdot L_{I}\right)}^{2}\left[4{\left(\frac{\varepsilon_{1}^{2}R_{1}^{3}}{\left(2+\varepsilon_{1}^{2}\right)\left(1-\varepsilon_{1}^{2}\right)c_{1}^{2}}+\frac{\varepsilon_{1}^{2}c_{1}^{2}R_{2}^{3}}{\left(2c_{2}^{2}+\varepsilon_{1}^{2}c_{1}^{2}\right)\left(c_{2}^{2}-\varepsilon_{1}^{2}c_{1}^{2}\right)}\right)}^{2}+\right.\\ \; & \left.\pi^{2}{\left(\frac{\varepsilon_{1}R_{1}^{3}}{\left(2+\varepsilon_{1}^{2}\right){\left(1-\varepsilon_{1}^{2}\right)}^{1/2}\;c_{1}^{2}}+\frac{\varepsilon_{1}c_{1}R_{2}^{3}}{\left(2c_{2}^{2}+\varepsilon_{1}^{2}c_{1}^{2}\right){\left(c_{2}^{2}-\varepsilon_{1}^{2}c_{1}^{2}\right)}^{1/2}}\right)}^{2}\right] \end{array}$$

In order to solve the equation $F(\omega ,\varepsilon_{1})=0$, it is necessary to assume numerical values defining the geometric parameters of the damper, the weight of the inertia ring, and the dynamic viscosity of the silicone oil. For the damper subjected to testing, the following values were assumed for the above parameters:

- the average inner radius $R_{1}=80.006$ mm;
- the average outer radius $R_{2}=109.922$ mm;
- the internal radial clearance $c_{1}=0.342$ mm;
- the external radial clearance $c_{2}=0.394$ mm;
- the height of the inertia ring $L_{I}=33.000$ mm;
- the weight of the inertia ring Q=44.512 N;
- the dynamic viscosity of the silicone oil $\eta_{10k}=9.76$, $\eta_{5k}=4.88$, $\eta_{2k}=1.952$, $\eta_{1k}=0.976$, $\eta_{500}=0.488$ i $\eta_{250}=0.244$ kg·m ^−1^s^−1^.

## 4. Results

The aim of the conducted research was to demonstrate that there is a certain critical viscosity of silicone oil at which the minimum height of the oil film corresponds to the surface roughness of the housing and inertia ring surfaces. For this reason, the tests were conducted from the highest to the lowest viscosity. After performing the test for a given oil viscosity, the damper was disassembled, the contact surfaces were inspected, and oil with a lower viscosity was added. The goal of the research was to find the viscosity level of silicone oil at which the inner surface of the inertia ring comes into contact with the surface of the housing.

### 4.1. Measurement of the Relative Angular Velocity of the Housing and the Inertia Ring

Experiments were conducted on the test stand with a damper filled with silicone oil of nominal kinematic viscosities $\nu_{10k}=0.01$, $\nu_{5k}=0.005$, $\nu_{2k}=0.002$, $\nu_{1k}=0.001$, $\nu_{500}=0.000500$, and $\nu_{250}=0.000250$ m^2^s^−1^ (corresponding to oils with viscosities of 10,000, 5000, 2000, 1000, 500, and 250 cSt). The tests were carried out at a temperature of 25 °C, at which the density of the silicone oil is 976 kg·m^−3^, and the corresponding dynamic viscosities are $\eta_{10k}=9.76$, $\eta_{5k}=4.88$, $\eta_{2k}=1.952$, $\eta_{1k}=0.976$, $\eta_{500}=0.488$, and $\eta_{250}=0.244$ kg·m^−1^s^−1^. The aim of the experiment was to determine the relative angular velocity ω between the housing and the inertia ring for a rotational speed of 1200 rpm and a compressor load of 10^6^ Pa.

**Figure 8:**
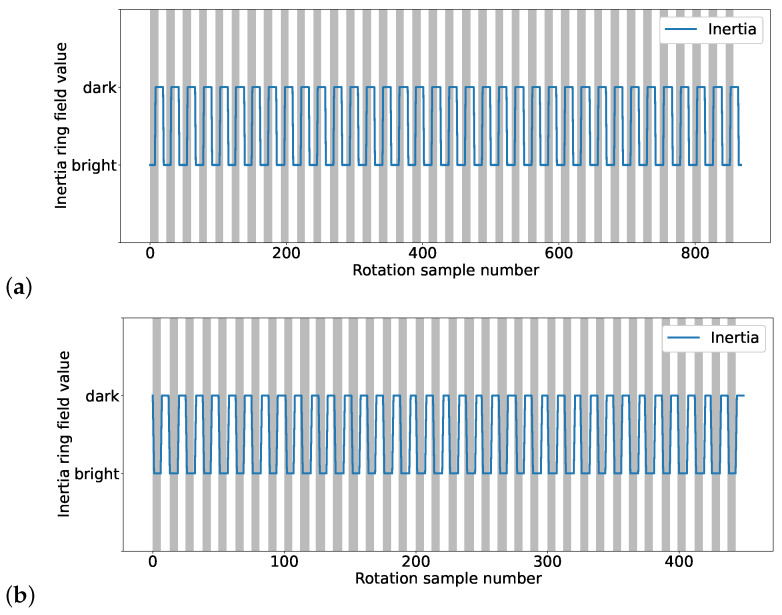
Sample graphs of the registration of light and dark fields of the housing (white and gray background stripes) and the inertia ring (blue graph) for silicone oil with a nominal kinematic viscosity of (**a**) $\nu_{10k}$ and (**b**) $\nu_{250}$.

During the experiments, infrared sensors recorded the presence of light and dark fields both on the housing and on the inertia ring. In Figure 8, sample readings from the first full rotation are presented for the damper filled with silicone oil of viscosity $\nu_{10k}$ (a) and oil with viscosity $\nu_{250}$ (b). The alternating gray and white stripes indicate the readings of the housing fields. The blue graph signals the readings of the inertia ring fields. A single rotation was considered during the tests as 36 dark fields and 36 light fields of the housing. These served as a reference point for reading the change in the position of the inertia ring.

**Figure 9:**
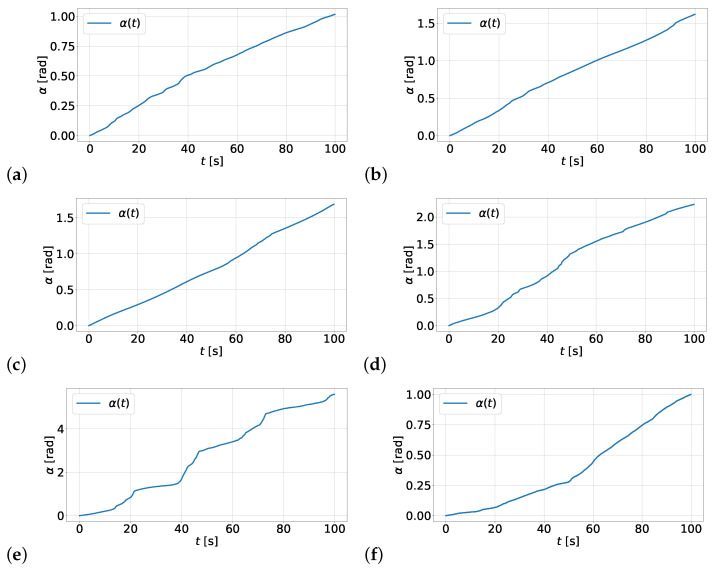
Graphs of the dependence of the relative angular displacement α of the housing and the inertia ring as a function of time for silicone oil with nominal kinematic viscosities of (**a**) $\nu_{10k}$, (**b**) $\nu_{5k}$, (**c**) $\nu_{2k}$, (**d**) $\nu_{1k}$, (**e**) $\nu_{500}$, (**f**) $\nu_{250}$.

Within the experiment, the relative displacement of the housing and inertia ring was registered as an angle α(t), where *t* represents the operating time of the test stand. The measurement results for the damper filled with various types of oil are presented in Figure 9. The graphs show the relative rotation of the housing and inertia ring for the damper filled with silicone oil with the following viscosities: (a) $\nu_{10k}$, (b) $\nu_{5k}$, (c) $\nu_{2k}$, (d) $\nu_{1k}$, (e) $\nu_{500}$, (f) $\nu_{250}$. The presented graphs indicate relatively stable angular velocity values for viscosities $\nu_{10k}$, $\nu_{5k}$, $\nu_{2k}$, and $\nu_{1k}$. In the case of the oil with viscosity $\nu_{500}$, the very unstable operation of the damper is observed, with a large range of angular velocity variation. However, all graphs from (a) to (e) show a systematic increase in the relative angular displacement between the inertia ring and the housing. In the case of the last test, presented in graph (f), there is a sharp drop in the change in angular displacement.

Based on the measurements performed, the minimum, maximum, and average angular velocities with which the inertia ring moved relative to the housing were determined. The numerical values of these velocities are presented in Table 4. Subsequent studies showed a systematic increase in the average angular velocity as the viscosity of the silicone oil decreased. However, for the oil $\nu_{250}$, there was a sudden drop in angular velocity, indicating the onset of dry friction between the surface of the inertia ring and the housing of the damper.

Additionally, in order to quantitatively illustrate the variability in the instantaneous angular velocity and the measurement uncertainty within a single experiment, Table 5 presents the basic descriptive statistics of the angular velocity ω(t) determined from the complete time series recorded for each investigated oil viscosity. The table reports the number of samples used in the analysis, the mean value $\bar{\omega }$, the standard deviation σ, and the coefficient of variation CV, which serves as a measure of the relative dispersion of the angular velocity.

### 4.2. Results of Numerical Simulations Based on the Determined Angular Velocity

**Figure 10:**
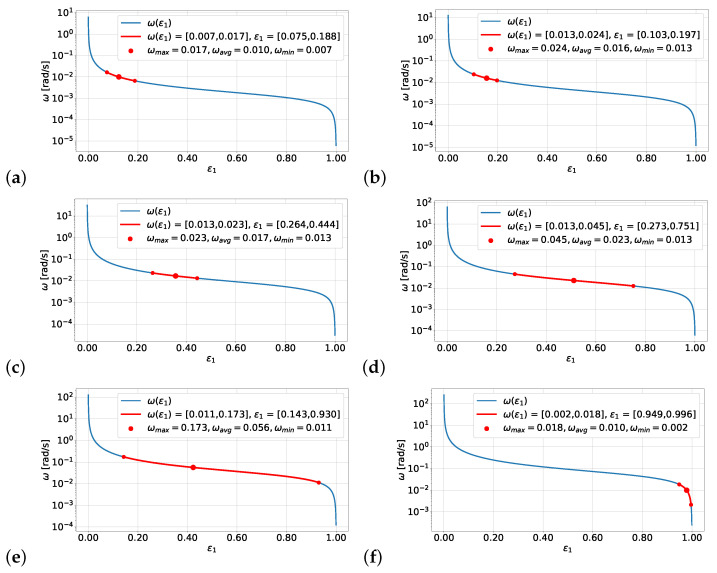
Graphs of the dependence of the relative angular velocity ω of the housing and the inertia ring as a function of the relative eccentricity $\varepsilon_{1}$ for silicone oil with nominal kinematic viscosities of (**a**) $\nu_{10k}$, (**b**) $\nu_{5k}$, (**c**) $\nu_{2k}$, (**d**) $\nu_{1k}$, (**e**) $\nu_{500}$, (**f**) $\nu_{250}$.

The numerical values of the minimum, maximum, and average angular velocity obtained during the tests were used to determine the relative eccentricity between the inertia ring and the housing. In Figure 10, plots of the function $\omega (\varepsilon_{1})$, calculated using Formula (4) and the measured quantities, are presented. It should be emphasized that the vertical axis is presented on a logarithmic scale to better visualize the obtained results. The theoretical course of the dependency $\omega (\varepsilon_{1})$, marked in blue, appears almost identical across all plots. However, attention should be paid to the scale, which differs between the plots and indicates different ranges on the vertical axis.

Graphs (a–c) show the device operating with high stability. The range of angular velocity variability is small, and the relative eccentricity $\varepsilon_{1}$ is greater than 0.5. In this context, the results for the damper filled with oils with viscosities of $\nu_{10k}$ and $\nu_{5k}$ appear particularly stable. Plot (d) also indicates proper device operation, but the range of angular velocities and relative eccentricities is much larger. Considering the exponential reduction in viscosity with temperature, these operating conditions should be considered inadequate. A temperature increase of several tens of degrees could shift the working range towards higher eccentricities.

Plot (e) illustrates the unstable operation of the device across a very broad range of angular velocities and relative eccentricities. This is a state where even small temperature changes can lead to the onset of dry friction between the working surfaces of the inertia ring and the casing. Meanwhile, plot (f) indicates that relative motion between the inertia ring and the housing has ceased. This means that the two working surfaces are in contact, resulting in dry friction.

### 4.3. Analysis of the Impact of Using Low-Viscosity Oil on the Contact Surfaces of a Viscous Damper

**Figure 11:**
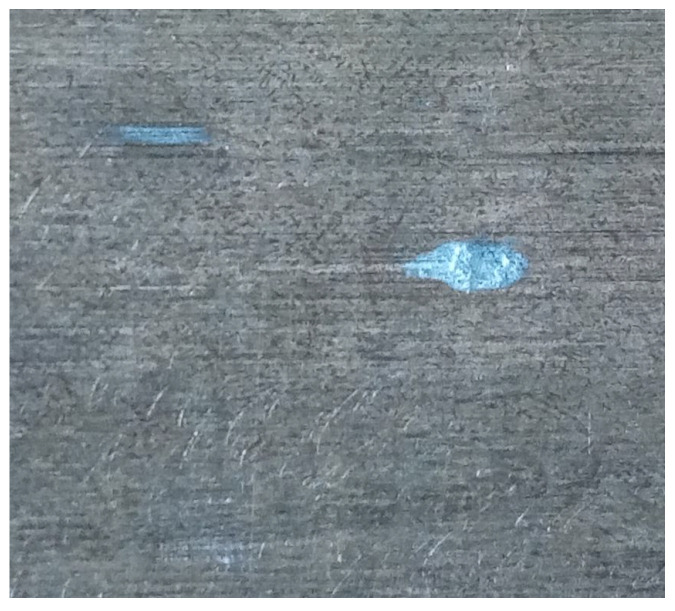
View of the contact surface of the inertia ring with visible material transfer due to adhesive wear.

**Figure 12:**
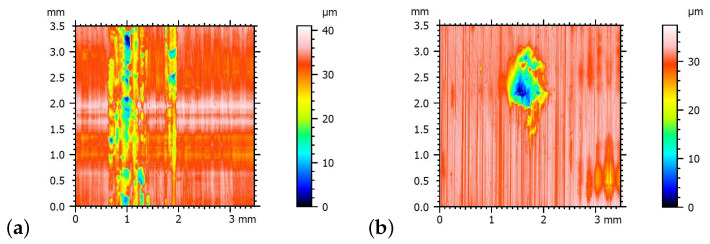
Topographic maps of the contact surfaces: (**a**) housing, (**b**) inertia ring, with visible signs of wear.

The use of oil with a viscosity of $\nu_{250}$ led to a decrease in the damping capacity of the viscous damper. As a result, both cooperating surfaces came into contact. This can be confirmed by analyzing surface wear, where the effects of contact are visible in the form of adhesive wear, evidenced by discoloration observed on the contact surface of the inertia ring (Figure 11), as well as deep grooves formed due to the transfer of material onto the cooperating surface of the damper housing, shown in Figure 12a. In both cases, the effects of larger wear products can be observed, which are detached from the surfaces of the material of both the inertia ring and the cooperating housing. This resulted in indentations that are also visible on the surface of the ring, shown in Figure 12b).

## 5. Discussion

The results of the conducted experiments demonstrate how the behavior of the inertia ring changes depending on the viscosity of the oil filling the damper. Although the experiments were conducted at a constant temperature (the operating time of the device was insufficient to cause significant heating), the obtained results also provide insight into what happens when the damper is heated—for example, from 25 °C to 80 °C. It is found that, in the case of PDMS, such a temperature increase leads to approximately a 2.5-fold decrease in viscosity [39]. This leads to the conclusion that the silicone oil filling the damper must have a viscosity at 25 °C that ensures stable operating conditions even after heating. It should be recalled that all experiments were conducted within a temperature range of 22–26 °C. Consequently, the damper behavior at other operating temperatures has not been experimentally validated and is inferred solely from the corresponding temperature-dependent changes in PDMS viscosity.

The experiments were conducted at a rotational speed of 1200 rpm and a constant compressor load. This operating point was selected as representative for typical working conditions of medium-sized diesel engines, such as those used in power generators, heavy-duty vehicles, and buses, which often operate at nearly constant rotational speeds. Additionally, 1200 rpm was the highest rotational speed at which stable measurements could be safely performed without risking damage to the experimental setup. While the present study was therefore limited to a single excitation frequency and load, the observed trends are expected to remain qualitatively similar at other operating conditions, with quantitative differences resulting primarily from changes in angular velocity amplitude and hydrodynamic loading.

It is also important to note how the minimum oil film thickness changes depending on the applied PDMS viscosity and relative angular velocity. For a typical viscous damper, the internal radial clearance is much smaller than the external clearance. Therefore, the minimum oil film thickness can be expressed by the following formula:

(5) $$\begin{array}{c} h_{\min,1}\left(\nu ,\omega \right)=\left(1-\varepsilon_{1}\left(\nu ,\omega \right)\right)\left(R_{I,1}-R_{H,1}\right)=\left(1-\varepsilon_{1}\left(\nu ,\omega \right)\right)c_{1}. \end{array}$$

A summary of the minimum oil film thicknesses for the tested viscosities, as well as the maximum, average, and minimum observed angular velocities, is presented in Table 6.

The values determined based on the model clearly show that, for the oil with a viscosity of $\nu_{250}$, there is direct surface contact and the occurrence of dry friction. The minimum film thicknesses for both the average and minimum speeds are lower than the sum of the Rzx parameters for both surfaces, which is 11.09 μm. The occurrence of surface contact and dry friction for this oil viscosity was also confirmed by the presence of adhesive wear. Furthermore, it should be noted that, for the oil with a viscosity of $\nu_{500}$, the minimum oil film thickness leaves a small margin relative to the sum of the Rzx parameters. The result is unstable damper operation, which, in the longer term, would likely lead to contact between the two surfaces.

Based on the predicted minimum oil film thickness $h_{1,\min}$, the maximum shear rate in the load-carrying lubricant layer was estimated using a Couette-type approximation $\dot{\gamma }\approx \omega R_{1}/h_{1,\min}$, where $R_{1}$ is the mean radius of the inner hydrodynamic layer. Using the minimum experimentally observed angular velocities for each oil viscosity, the estimated shear rates were ${\dot{\gamma }}_{10k}=1.89\;s^{-1}$, ${\dot{\gamma }}_{5k}=3.64\;s^{-1}$, ${\dot{\gamma }}_{2k}=5.59\;s^{-1}$, ${\dot{\gamma }}_{1k}=11.77\;s^{-1}$, ${\dot{\gamma }}_{500}=37.58\;s^{-1}$, and ${\dot{\gamma }}_{250}=141.94\;s^{-1}$. As reported in the technical documentation of Bayer AG [55], for silicone oils with kinematic viscosities below 0.01m^2^·s^−1^, non-Newtonian effects occur only at shear rates exceeding approximately 1000s^−1^, which is well above the shear rates estimated in the present study.

To quantitatively assess the sensitivity of the model results to uncertainty in the angular velocity measurement, a local analysis of the relationship between variations in the relative eccentricity $\varepsilon_{1}$ and angular velocity ω was performed. For the implicitly defined equilibrium relation $F(\omega ,\varepsilon_{1})=0$, small perturbations satisfy the relation

(6) $$\begin{array}{c} \Delta \varepsilon_{1}=a\left(\omega ,\varepsilon_{1}\right)\Delta \omega , \end{array}$$

where

(7) $$\begin{array}{c} a\left(\omega ,\varepsilon_{1}\right)=-\frac{\partial F}{\partial \omega }{\left(\frac{\partial F}{\partial \varepsilon_{1}}\right)}^{-1}. \end{array}$$

This formulation allows the uncertainty in ω to be directly propagated to uncertainty bounds for $\varepsilon_{1}$. The numerically determined sensitivity coefficients and the resulting eccentricity uncertainties for all investigated oil viscosities are summarized in Table 7. The table reports the numerically evaluated sensitivity coefficient $a(\omega ,\varepsilon_{1})$ and the resulting uncertainty $\Delta \varepsilon_{1}$ determined from the standard deviation σ of the measured averaged angular velocity $\omega_{\mathrm{avg}}$.

The proposed model is not limited to the specific damper geometry investigated in this study and can be generalized to other viscous damper configurations. The key requirement for such generalization is the identification of load-carrying lubricant layers that can be treated independently within the hydrodynamic lubrication framework.

If the damper geometry can be described by a set of mean radii $R_{1},\ldots ,R_{n}$, corresponding radial clearances $c_{1}<\ldots <c_{n}$, and relative eccentricities $\varepsilon_{1},\varepsilon_{2}=\frac{c_{1}}{c_{2}}\varepsilon_{1},\ldots ,\varepsilon_{n}=\frac{c_{1}}{c_{n}}\varepsilon_{1}$, the equilibrium formulation can be extended accordingly. In such cases, Equation (4) may be modified to include the resultant hydrodynamic supporting force obtained as the superposition of contributions from an arbitrary number of lubricant layers.

Consequently, the model can be applied to dampers with more complex geometries or multi-gap configurations, provided that the hydrodynamic pressure distributions in individual layers remain weakly coupled and the underlying assumptions of a laminar flow and hydrodynamic lubrication are satisfied.

Unlike conventional rheological or tribological studies performed under controlled laboratory conditions, the present approach characterizes PDMS through its dynamic interaction with a mechanical system. This system-level perspective enables the identification of functional operating limits of PDMS under real hydrodynamic loading, which are directly relevant to torsional vibration damper applications. In this sense, the study extends existing PDMS characterization beyond material testing toward in situ functional assessment.

## 6. Conclusions

This study presents experimental and model-based results on the behavior of a torsional vibration viscous damper filled with PDMS oils of different viscosities. The dedicated test rig and optical measurement method enabled the identification of characteristic damper operating regimes as the viscosity decreased, including stable operation, unstable behavior with large fluctuations in the relative angular velocity, and the transition to boundary/dry friction evidenced by surface damage. The results show that an excessively low oil viscosity leads to the loss of hydrodynamic separation and contact between the working surfaces, whereas the admissible minimum viscosity depends strongly on the surface condition (roughness) and the internal clearance. The observed trends are consistent with the hydrodynamic lubrication-based model, supporting its use in predicting operating regime transitions and assisting in the selection of the damper geometry and PDMS viscosity in relation to the required lubrication stability.

## Figures and Tables

**Table 1:** Damper housing material.

Property	Value
Material	PA6/2017/AlCu4MgSi(A)
Density [kg·m^−3^]	2.82 $\times \;10^{3}$
Thermal expansion coefficient [K^−1^]	2.30 ×10^−5^
Thermal conductivity [W·m^−1^K^−1^]	140 ÷ 180
Tensile strength (Rm) [Pa]	≥3.70 × 10^8^
Yield strength (Rp 0.2) [Pa]	≥2.15 × 10^8^
Elongation (A) [%]	≥15
Brinell hardness (HB)	60 ÷ 110

**Table 2:** Inertia ring material.

Property	Value
Material	Bronze B101/CuSn10P
Density [kg·m^−3^]	8.90 $\times \;10^{3}$
Thermal expansion coefficient [K^−1^]	1.70 ×10^−5^
Thermal conductivity [W·m^−1^K^−1^]	57
Tensile strength (Rm) [Pa]	≥2.00 × 10^8^
Yield strength (Rp 0.2) [Pa]	≥1.00 × 10^8^
Elongation (A) [%]	≥4
Brinell hardness (HB)	60 ÷ 100

**Table 3:** Viscous fluid filling the damper during the experiments.

Property	Value
Material	PDMS
Flash point [°C]	>300
Density in 25 °C [kg·m^−3^]	9.76 $\times \;10^{2}$
Thermal expansion coefficient [K^−1^]	$9.5\times \;10^{-6}$
**Kinematic Viscosity in 25 °C [m^2^s^−1^]**	
Experiment number 1 for $\nu_{10k}$	$1.0\times 10^{-2}$
Experiment number 2 for $\nu_{5k}$	$5.0\times 10^{-3}$
Experiment number 3 for $\nu_{2k}$	$2.0\times 10^{-3}$
Experiment number 4 for $\nu_{1k}$	$1.0\times 10^{-3}$
Experiment number 5 for $\nu_{500}$	$5.0\times 10^{-4}$
Experiment number 6 for $\nu_{250}$	$2.5\times 10^{-4}$

**Table 4:** Minimum, maximum, and average angular velocities of the inertia ring relative to the housing, calculated over the entire measurement period.

Oil Viscosity Viscosity	Minimum Angular Velocity over Entire Test [$10^{-3}\cdot$ rad·s^−1^]	Maximum Angular Velocity over Entire Test [$10^{-3}\cdot$ rad·s^−1^]	Average Angular Velocity over Entire Test [$10^{-3}\cdot$ rad·s^−1^]
$\nu_{10k}$	6.575	16.519	10.055
$\nu_{5k}$	12.523	24.001	15.935
$\nu_{2k}$	13.320	23.214	16.939
$\nu_{1k}$	12.532	44.818	22.560
$\nu_{500}$	11.226	173.058	56.163
$\nu_{250}$	2.129	18.458	9.924

**Table 5:** Statistical descriptors of the relative angular velocity ω determined from the complete time series recorded during a single experiment for each oil viscosity.

Oil Viscosity	Number of Samples	$\bar{\omega }$ [$10^{-3}\cdot$ rad·s^−1^]	σ [$10^{-3}\cdot$ rad·s^−1^]	$CV=\bar{\omega }/\sigma$
$\nu_{10k}$	143	10.185	3.546	0.348
$\nu_{5k}$	143	16.209	3.678	0.227
$\nu_{2k}$	143	16.876	3.300	0.196
$\nu_{1k}$	144	22.313	10.781	0.483
$\nu_{500}$	144	55.815	54.108	0.969
$\nu_{250}$	144	9.990	5.571	0.558

**Table 6:** Summary of the minimum oil film thicknesses $h_{\min,1}\left(\nu ,\omega \right)$. Surface contact is assumed when $h_{\min,1}$ falls below the roughness threshold defined as the sum of the Rzx parameters of both surfaces, $R_{zx,\mathrm{housing}}+R_{zx,\mathrm{inertia}\;\mathrm{ring}}=11.09\;\mu$m.

Oil Viscosity	$h_{\min,1}(\cdot ,\omega_{\max})$ [μm]	$h_{\min,1}(\cdot ,\omega_{\mathrm{avg}})$ [μm]	$h_{\min,1}(\cdot ,\omega_{\min})$ [μm]
$\nu_{10k}$	316.8	300.3	278.2
$\nu_{5k}$	307.1	289.3	275.0
$\nu_{2k}$	252.1	220.4	190.6
$\nu_{1k}$	249.0	167.5	85.2
$\nu_{500}$	293.5	197.5	23.9
$\nu_{250}$	17.5	7.2	1.2

**Table 7:** Sensitivity analysis of the relative eccentricity $\varepsilon_{1}$ with respect to angular velocity measurement uncertainty.

Oil Viscosity	$\omega_{\mathrm{avg}}$ [$10^{-3}\cdot$ rad·s^−1^]	$\varepsilon_{1}(\omega_{\mathrm{avg}})$	$a(\omega ,\varepsilon_{1})$ **[rad^−1^·s]**	σ [$10^{-3}\cdot$ rad·s^−1^]	$\Delta \varepsilon_{1}=a\cdot \sigma$
$\nu_{10k}$	10.055	0.123	−12.187	3.546	−0.043
$\nu_{5k}$	15.935	0.155	−9.654	3.678	−0.035
$\nu_{2k}$	16.939	0.356	−19.652	3.300	−0.065
$\nu_{1k}$	22.560	0.511	−18.564	10.781	−0.200
$\nu_{500}$	56.163	0.423	−6.740	54.108	−0.365
$\nu_{250}$	9.924	0.979	−2.784	5.571	−0.015

## Data Availability

The original contributions presented in this study are included in the article. Further inquiries can be directed to the corresponding author.
